# Generalized Extension of Referred Trigeminal Pain due to Greater Occipital Nerve Entrapment

**DOI:** 10.1155/2023/1099222

**Published:** 2023-11-11

**Authors:** Jung-woo Hyung, Byung-chul Son

**Affiliations:** ^1^Department of Neurosurgery, Seoul St. Mary's Hospital, College of Medicine, The Catholic University of Korea, Seoul, Republic of Korea; ^2^Catholic Neuroscience Institute, College of Medicine, The Catholic University of Korea, Seoul, Republic of Korea

## Abstract

We report a very rare case of referred pain caused by greater occipital nerve (GON) entrapment, inducing spontaneous pain in the whole body as well as in the trigeminal nerve region of the face and head. It has already been reported that entrapment of the GON can induce referred pain in the ipsilateral limb as well as the ipsilateral hemiface. A 42-year-old female patient presented with chronic pain in her gums, jaw angle, submandibular region, retro-auricular suboccipital, and temporo-occipital vertex that had been ongoing for four years. As the patient's head pain and facial pain became severe, severe spontaneous pain occurred in the arm, waist, and both lower extremities. This patient's pain in the occipital and neck, spontaneous pain in the face, jaw, and whole body improved with decompression of the GON. Anatomical basis of pain referral to the facial trigeminal area caused by chronic GON entrapment is convergence of nociceptive inflow from high cervical C1–C3 structures and trigeminal orofacial area in the dorsal horn of the cervical spinal cord from the C2 segment up to the medullary dorsal horn (MDH). The major afferent contribution among the suboccipital and high cervical structure is mediated by spinal root C2 that is peripherally represented by the GON. Chronic noxious input from GON entrapment can cause sensitization and hypersensitivity in second order neurons in the trigeminocervical complex (TCC) and MDH in the caudal trigeminal nucleus and high cervical cord. Generalized extension of referred pain due to GON entrapment is thought to involve two possible pathophysiologies. One is the possibility that generalized pain is caused by sensitization of third-order nociceptive neurons in the thalamus. Another speculation is that spontaneous pain may occur throughout the body due to dysfunction of the descending brain stem pain-modulating pathway by sensitization and hyperexcitation of the MDH and trigeminal brainstem sensory nuclear complex (TBSNC).

## 1. Introduction

Occipital neuralgia is defined as a unilateral or bilateral paroxysmal, shooting, or stabbing pain in the posterior part of scalp with distribution of greater and lesser occipital nerves (GON, LON) [1]. Excluding structural lesions involving craniovertebral junction that can compress or irritate the C2 cervical root or GON, occipital neuralgia has been mostly considered to have an idiopathic etiology [2]. However, entrapment of the GON within the trapezial tunnel at its tendinous aponeurotic attachment to the superior nuchal line has been found to be the main cause of occipital neuralgia based on anatomical studies and results of GON decompression surgeries for occipital neuralgia and occipital-based headaches [3–8]. Occipital neuralgia is well known as a symptom of GON entrapment. However, paroxysmal stabbing pain of occipital neuralgia is not the only symptom of GON entrapment. GON entrapment commonly induces continuous aching, tightening, and pressure-like pain in the GON distribution, as well as intermittent stabbing pain in typical occipital neuralgia [7–9].

The nociceptive afferent inflow from occipital and suboccipital structures is mediated by small-diameter afferent fibers in the upper cervical roots terminating in the dorsal horn of the cervical cord extending from the C2 segment up the medullary dorsal horn of the caudal trigeminal nucleus [10]. The major afferent contribution is mediated by the C2 spinal root that is peripheral represented by the GON [11]. The GON originates in the medial branch of the dorsal rami of the second cervical root and transmits nociceptive afferent information from the temporo-occipital and vertex as well as the suboccipital area [12]. The GON shows a high convergence of input from neck muscle and the skin [13]. Therefore, the GON constitutes the main sensory afferent nerve through the C2 root. This afferent input is transmitted directly the C2 dorsal horn [13, 14].

Nociceptive afferents from high cervical region converge with that of the trigeminal system by the second-order nociceptive neurons of the trigeminocervical complex (TCC) in the caudal trigeminal nucleus and upper cervical cord [13, 14]. The term “trigeminocervical complex” encompasses the caudal spinal trigeminal nucleus and laminae I-II of the upper 2 cervical spinal segments, the commissural division of the nucleus of the solitary tract, and its caudal extension into spinal lamina X [15]. It is where electrical stimulation of the middle meningeal artery or superior sagittal sinus induced upregulation of c-Fos expression in cats and monkeys [16]. These dura-sensitive, second-order trigeminal nociceptive neurons in the TCC show a high degree of convergent input from other trigeminal afferents such as facial and corneal areas [17].

Convergence of nociceptive trigeminal and occipital afferent input in the spinal trigeminal nucleus and dorsal horn of the upper cervical cord is an anatomical substrate for central sensitization [10, 13–17]. Convergence along with sensitization of the TCC neurons in the brain stem and upper spinal cord provides a physiological basis for the clinical phenomenon of spread and referred pain between trigeminal and cervical areas by which pain originating from an affected tissue is perceived as originating from a distant receptive field [14]. In a rat model of chronic constriction injury of the infraorbital nerve, stimulation of the GON reduced the response of TCC neurons to light mechanical stimuli and this modulatory effect was found to be mediated by GABAergic and glycinergic mechanisms [18, 19].

It has been reported in recent years that chronic noxious afferent input from entrapment can result in sensitization and hypersensitivity of secondary neurons in the TCC, resulting in referred pain in the facial trigeminal distribution [20–26]. Referral to the orofacial area occurs not only in the V1 (ophthalamic) region but also in V2 (maxillary) and V3 (mandibular) regions. It even causes hemifacial sensory changes [20, 21]. Referred pain from GON entrapment has been reported to cause deep ear pain [24]. It might also extend to the ipsilateral arm and leg beyond the cranio-cervical region [26]. It has been confirmed that the referred facial trigeminal pain caused by GON entrapment can disappear with decompression of the GON [20–26]. This report shows that referred pain with central sensitization due to GON entrapment can extend not only to the trigeminal nociceptive area but also to the whole body, which is the area of spinal nociception.

## 2. Case Report

**Figure 1:**
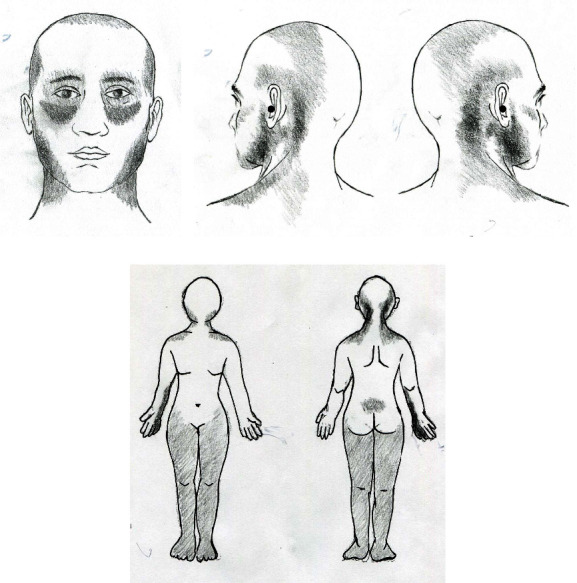
Schematic drawing showing the distribution of characteristics of chronic pain. (a) The grey areas over bilateral periorbital, gums, malar, mental, and submental area indicate the distribution of pain that occurred 4 years ago. The nature of pain was throbbing and tightening, which lasted all day. (b) The location (gray areas) of temporal-occipital and parietal pain concurrent with facial and submental pain. It has also spread to the nape of the neck and shoulders. When the pain was severe, it was accompanied by a stabbing pain in the bilateral ear canal (black circles). (c) The location of spontaneous paresthesia in the right medial forearm and hand that occurred one year after onset of facial and occipital pain. Subsequently, spontaneous pain and numbness extended to the lower back and both legs.

A 42-year-old female patient presented with chronic pain in her gums, jaw angle, submandibular region, retro-auricular suboccipital, and temporo-occipital vertex that had been ongoing for four years. The patient reported that throbbing and tightening pain gradually developed on both sides of her gums, at the angle of the jaw, and under the jaw four years ago (Figure 1). At the same time, pain of the same nature occurred in the suboccipital and temporal region. In fact, six years before the onset of pain in the head and face, she had been suffering from aching pain in both the nape of the neck and shoulders (Figure 1). The patient was treated by a dentist for dental caries. However, her symptoms did not improve despite the treatment. She visited several other dentists, who informed her that there was no problem with her existing caries treatment. She also received treatment for inflammation of the parotid gland at the otolaryngology department of a university hospital due to pain under the jaw. However, there was no improvement. Finally, the patient was diagnosed with temporomandibular joint disease and treated by a dentist at another university hospital. Again, her symptoms did not improve.

The pain in her both molars, gums and jaw, and retro-auricular suboccipital area lasted 24 hours relentlessly. When the pain was severe, it was accompanied by a stabbing pain in the bilateral ear canal. No abnormality was found at otolaryngologic examinations. For her nape and neck pain, physical therapy and multiple medications were administered for more than two years under the diagnosis of tension headache and degenerative cervical spine disease. Several conventional NSAIDs (including Ultracet® and ibuprofen) and drugs such as gabapentin, pregabalin, and muscle relaxants had no effect at all. One year after the onset of jaw and occipital pain, paresthesia suddenly developed in her right forearm and hand for no apparent reason (Figure 1). It occurred at work for no specific reason, such as trauma or lifting objects. The numbness in her right arm also continued throughout the day, especially in the morning.

Pain and suffering in her gums, jaw, submandibular area, occipital, and neck, lasted for 4 years without interruption. They gradually worsened one year before admission. The throbbing and tightening pain in the retro-auricular suboccipital area extended to both temporal and vertex areas. At the same time, a foreign body sensation as if there was sand in the eye occurred in both eyes and stabbing pain occurred in both malar areas (Figure 1). A subjective feeling of blurred vision occurred due to periorbital foreign body sensation. She visited an ophthalmologist. However, no abnormalities were found and her vision was normal. If she was unable to sleep well due to pain in her jaw and head, tinnitus, like a beeping sound, occurred in her ears, along with stabbing pain in her ears. Because of her facial pain that occurred in addition to the jaw pain, her dentist ordered to wear a temporomandibular joint brace along with botulinum toxin injections. However, it did not help her pain. Severe jaw and facial pain accompanied by occipital pain forced her to quit her job. Stellate ganglion blocks and trigeminal nerve blocks performed at the pain clinic were ineffective.

At about two months after worsening of the periorbital and facial pain, existing numbness in the medial right forearm and right 4th and 5th fingers got worse for no reason. In addition, numbness and pain developed gradually in her back and both legs (Figure 1). They also occurred without a specific cause, such as trauma or hard work. She described the pain in her lower back as a tearing sensation. The pain in the lower back continued to exist regardless of movement or walking. Numbness in both legs was diffuse, including the feet, without dermatomal distribution. It also lasted for 24 hours. Along with her back pain, she reported experiencing a dull, aching pain in both knees, which made walking difficult. Computed tomography of the lumbar spine performed due to low back pain did not show any abnormal findings. Orthopedics and neurosurgeons confirmed that her knees and back were intact. Epidural block was performed four times. However, its effect did not last for more than 2 hours. Pain medications prescribed for back and knee pain did not help at all.

In addition to the pain in her molars and gums, jaw, and occipital area, which persisted for three years, pain in her eyes, cheeks, and back pain associated with numbness in her right hand and forearm and both legs made her unable to carry out her daily life. She was worried that there was something wrong with her head. So she underwent an MRI scan of the brain. However, nothing was wrong. Several neurologists diagnosed her with migraine or tension-type headache. However, they did not provide her a comprehensive explanation for the facial pain accompanying the headache. Medications prescribed for headaches did not work even after months of use.

She visited our outpatient clinic to see if she was suffering from trigeminal neuralgia, with systemic pain that began with her gums and jaw and expanded to her head and whole body. The chronic pain made her unbearable. Despite medication, physical therapy, and back and cervical injections, she rated her pain as severe with an NRS 7/10. She has been unable to work since a year ago and expressed her difficulty that she could hardly even do a home life.

**Figure 2:**
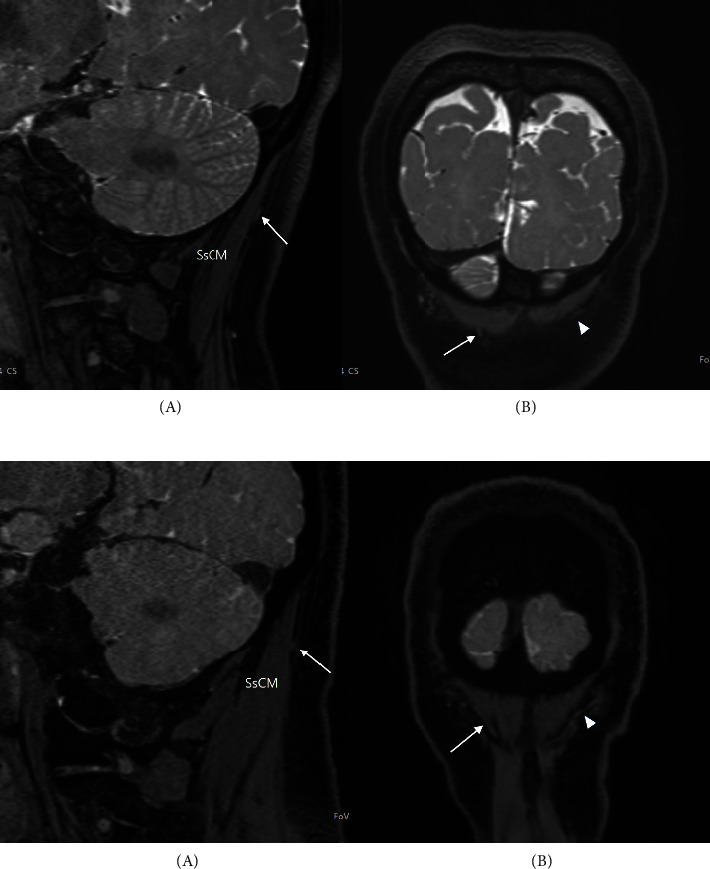
MRI images of the greater occipital nerve (GON). (a) Sagittal (A) and coronal (B) MR images of the right GON (white arrow) in the current case. The left image represents a sagittal T2-weighted image (t2_de3d_we,_iso 0.7 mm, MPR, Magnetom Terra, Siemens, Germany) of the right GON taken along the distal portion path within the trapezial tunnel. The image on the right is a coronal MR image (t2_de3d_we, iso 0.7 mm, MPR) showing the course of the right and left (white arrowhead) GONs within the trapezial tunnel. SsCM; semispinalis capitis muscle. (b) Sagittal (A) and coronal (B) MR images of the right GON (white arrow) taken with the same MR sequence (t2_de3d_we,_iso 0.7 mm, MPR) in a patient suffering from right trigeminal neuralgia. This is presented for comparison with images from patients with GON entrapment. The image on the right is a coronal MR image (t2_de3d_we, iso 0.7 mm, MPR) showing the course of the right and left (white arrowhead) GONs within the trapezial tunnel. This MRI image shows that it is still difficult to clearly diagnose GON entrapment even with high-resolution MRI. SsCM, semispinalis capitis muscle.

Despite pain and discomfort in her face and neck, no objective sensory change in her face was confirmed. No neurological abnormalities were detected including masticatory function in the trigeminal nerve, other cranial nerves, or extremities. No allodynia or tenderness was found in the occiput, neck, or back. There were no movement restrictions involving the neck or back. No physical findings suggestive of cervical radiculopathy were found. There were no restrictions on the straight leg raising test of the lower limbs. No abnormalities were found on physical examination of the pelvis or legs including the hip joint. Laboratory examinations showed normal findings, including an erythrocyte sedimentation rate (ESR) and uric acid. Magnetic resonance imaging (MRI) scans suspecting trigeminal neuropathy did not show vascular compression or abnormal enhancement of the trigeminal nerve. No abnormalities were identified in the path and signal intensity of the GON in suboccipital space (Figure 2). No abnormalities were detected in the computed tomographic (CT) scan of the cervical spine performed to check for structural lesions in the path from the C2 nerve root to the GON. There was no medical history including hypertension or diabetes except chronic intermittent neck and shoulder pain.

The patient's facial and temporo-occipital pain was presumed to be a referred facial trigeminal pain due to chronic GON entrapment. This was because the authors had already experienced many cases of suddenly appearing referred trigeminal pain in the face accompanied by occipital pain in many patients with GON entrapment. In addition, pain within ipsilateral ear canal and lateral neck was frequently reported by patients with GON entrapment. It has been reported that pain associated with GON entrapment radiates not only to the occipital area, but also to the temporal, vertex, retro-auricular and subauricular neck areas, angle of the jaw, and lateral and posterior neck. The pain location in the submandibular area of the current case, which was difficult to explain, coincided with subauricular and submandibular areas known to be pain distribution areas of GON entrapment [18–24]. In addition, it has been reported that referred pain of chronic GON entrapment might extend not only to trigeminal distribution but also the arms and legs [26].

With the possibility of referred facial pain due to GON entrapment in mind, occipital nerve block (ONB) of bilateral GON was performed using 2 mL of 2% lidocaine, which relieved the intensity of occipital, periorbital, and malar pain by 80% for 2 hours. The same temporary improvement was confirmed in the second block of bilateral GONs. The second ONB temporarily alleviated the pain in the right arm and back pain as well as occipital pain for 1 hour. Three hours after receiving ONB, pain in the face, neck, and arm continued to persist with the same intensity as before. Due to chronicity and refractoriness of the pain and possibility of referred facial pain due to chronic entrapment of GON, decompression of the GON was recommended after obtaining written informed consent.

**Figure 3:**
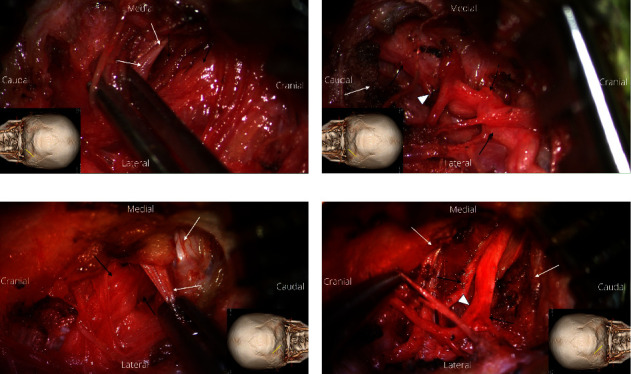
Intraoperative images showing the entrapment of the greater occipital nerve (GON) during the decompression. (a) Intraoperative images showing severe entrapment of the right GON (black arrows) by the fibrous, aponeurotic edge of the trapezius muscle (white arrows) along the superior nuchal line. The inset shows the direction of the image and the location of the incision. (b) Intraoperative image after decompression of the right GON (black arrows) with division of the trapezial aponeurosis (white arrows). The most severe part of the GON constriction is indicated by a white arrowhead. (c) Intraoperative image showing an entrapment of the left GON (black arrows) with elevation of the aponeurotic edge of the trapezius muscle (white arrows). The inset shows the direction of the image and the location of the incision. (d) Intraoperative image after decompression of the left GON (white arrows) with division of the trapezial aponeurosis (white arrows). The maximal compression area (white arrowhead) of the left GON shows reddish discoloration and constriction.

The author's GON decompression technique using an oblique, paramedian approach using a microscope has already been reported [20–26]. In brief, it is a distal approach that first addresses the distal branch of the GON that has passed through the trapezial canal. Under general anesthesia, a 3-cm long, paramedian oblique incision was made bilaterally in the presumed course of the GON. While dissecting the subcutaneous tissue, a small self-retractor was applied by dissecting the subcutaneous tissue into 3 to 4 parts transversely, without damaging small branches of the GON using a microscope. Tracing the small visible branch of the GON in the subcutaneous tissue revealed the main branch of the GON, which was compressed against the tendinous aponeurotic edge of the trapezius (Figure 3). It was confirmed that this tendinous aponeurotic edge of the trapezius muscle was the main entrapment site of the GON. The aponeurotic edge was dissected and the proximal course of the GON was dissected circumferentially. The path was released by dissecting the semispinalis capitis muscle located under the trapezius to the point of GON emergence (Figure 3).

The effect of GON decompression appeared from the day after surgery. The patient reported that the persistent pain and discomfort in the bilateral periorbital, malar, jaw, and occipital area was reduced by about 80% compared to the previous condition. At the same time, it was reported that the tearing pain in the back and legs, and right arm paresthesia were no longer felt.

Two months after the operation, the pain in the back of the head, neck, and limbs was not severe. It did not interfere with her daily life. The pain in the jaw and cheek was not severe (NRS 3-4/10). However, it was often aggravated for 2-3 hours when she was tired or stressed. Medication continued to be necessary. However, the effect of the medication was insignificant. She reported that her back and knee pain was gone and that she was able to walk for more than 30 minutes. Now she could climb stairs without difficulty. The temporomandibular joint brace was also withdrawn. She was able to return to her job. Six months after surgery, the patient reported doing well without severe pain in the face or jaw. She said she no longer felt tearing pain in her back or leg or paresthesia in her right arm. She reported intermittent discomfort around her chin. However, no further medication was needed (NRS 2/10).

## 3. Discussion

### 3.1. The Greater Occipital Nerve and Its Connection to the Brain Stem

The GON originates from the medial branch of the dorsal rami of the second cervical root [27]. It also receives a branch from the dorsal rami of the C3 nerve [27]. It ascends through the semispinalis capitis muscle and runs posterolaterally before emerging into the scalp by piercing the aponeurotic fibrous sling between the trapezius and sternocleidomastoideus muscle near their attachment to the superior nuchal line [27, 28]. This aperture is a common site of GON entrapment. It was named a trapezial tunnel [4, 5]. After piercing the aponeurotic sling of the trapezial tunnel, the GON widens along its course to the periphery in contrast to other peripheral nerves [3–5, 28]. This finding is regarded as relevant to GON entrapment, as widening of the nerve increases its susceptibility to entrapment, especially in the firm trapezius aponeurosis [3]. The GON transmits nociceptive afferent information form the temporo-occipital and vertex as well as suboccipital area [3–5, 7, 8].

Upper cervical roots contribute to the sensory innervation of posterior head and neck [13, 14, 28, 29]. Occipital and suboccipital structures, such as vessels and the dura mater of the posterior dura, deep paraspinal neck muscles, zygapophyseal joints, and ligaments, are innervated by upper cervical roots. They are recognized sources of neck and head pain [13, 14]. The nociceptive inflow from these suboccipital structures is mediated by small-diameter afferent fibers in the upper cervical roots terminating the dorsal horn of the cervical cord extending from the C2 segment up to the medullary dorsal horn [13, 14]. Major afferent contribution is mediated by the C2 spinal root that is peripherally represented by the GON [13, 14, 29]. Therefore, the GON constitutes the main sensory afferent nerve through the C2 root. This afferent information is transmitted directly to the C2 dorsal horn [13, 14].

### 3.2. Convergence of Trigeminal and Occipital Nociceptive Afferents and Central Sensitization

Convergence of nociceptive trigeminal and occipital afferent input in the spinal trigeminal nucleus and dorsal horn of the upper cervical cord is an anatomical substrate for central sensitization [13–17, 30–33]. Noxious input from receptors on the distal end of the fifth nerve, which innervates the craniofacial tissue, passes through the trigeminal ganglion and then into the trigeminal tract. After entering the tract, most nociceptive afferents pass caudally while giving off collaterals that terminate in subdivisions of the spinal trigeminal nucleus and upper cervical cord to activate second-order neurons [33]. The spinal trigeminal sensory nucleus (Sp5) consists of three subnuclei (oralis, Sp5O; interpolaris, Sp5I; and caudalis, Sp5C) [33]. Sp5C is called the medullary dorsal horn because it is the only part with a layered structure and morphological and functional organization comparable to that of the spinal dorsal horn [30, 31, 33]. The great majority of nociceptive primary afferents terminates in superficial layers (laminae I and II), although some A*δ* fibers terminate in lamina V of Sp5C [33].

It has been shown that Sp5C trigeminovascular neurons receiving convergent input from dura and periorbital area not only increase their responses following application of inflammatory agents to dura but also become sensitized for several hours [17]. These sensitized Sp5C neurons have low thresholds to both dural and peri-ocular skin stimulation. They show a significant increase in the size of their dural and cutaneous receptive fields [17]. Based on these findings, it has been proposed that the referred, cutaneous allodynia observed in migraine patients is due to central sensitization of Sp5C neurons following peripheral sensitization of meningeal nociceptors [17]. In addition, the ophthalmic region of Sp5C, which contains neurons that receive convergent input from the dura and peri-orbital skin, sends projections to the ophthalmic primary afferent projection area of the contralateral trigeminal brain stem sensory complex [34, 35]. These projections are somatotopically organized, extending rostrocaudally from the caudal spinal trigeminal nucleus to the upper cervical dorsal horn C2–3 segments [34, 35]. Contralateral projections could provide input that specifically modulates the activity of medullary and spinal dorsal horn cells driven from the ophthalmic division of the trigeminal nerve [34, 35]. Such input could become effective following long-lasting noxious stimulation of meningeal nociceptors, thus contributing to central sensitization that occurs after long-lasting migraine attacks [32]. In migraine patients, Sp5C sensitization could elicit cutaneous allodynia that extends outside the referred pain area to the skin over the contralateral head and forearm [36].

Sp5C also projects to the ipsilateral junction of Sp5I, Sp5O, and principal sensory (Pr5) nuclei over their whole caudal–rostral extent [37]. Such intra-trigeminal connections are also somatotopically organized [37, 38]. Ipsilateral input from Sp5C neurons to rostral trigeminal nuclei could contribute to amplification of nociceptive output to supramedullary structures via interpolar, oral, and principal subdivisions since these regions convey orofacial input to the brain stem and thalamic areas [33].

Glutamatergic transmission is important for sensitization of second-order nociceptive, convergent neurons in the Sp5C [33]. Rostral trigeminal nuclei, especially Sp5O, can convey both extra- and intra-oral nociceptive input, which is dependent on glutamatergic input from Sp5C [37]. Following intense noxious stimulation or nerve injury, fine primary afferents can release glutamate and several other peptides and neuromodulators onto lamina I neurons [33, 37, 39]. When normally silent NMDA receptors become activated, they can lead to a cascade of calcium-dependent and second-messenger signaling that can increase the excitability of lamina I neurons and facilitate transmission of noxious messages to the brain [33, 39]. Under such circumstances, lamina I nociceptive neurons could also be activated by A*β* non-nociceptive primary afferents that usually drive inhibitory interneurons [33, 39]. Following injury, A*β* fibers could activate PKC-*γ*–expressing interneurons in inner lamina II, which become disinhibited and in turn activate lamina I neurons [33, 39, 40].

### 3.3. Pathophysiology of Extra-Trigeminal, Generalized Referred Pain from GON Entrapment

Considering that the referred pain due to GON entrapment extended to the back and limbs as well as the trigeminal nerve distribution of the face, it could be inferred that not only nociceptive second-order neurons in brain stem but also central sensitization involving third-order neurons were involved in the pathophysiology.

In pain disorders such as fibromyalgia, focal somatic pain might be associated with cutaneous allodynia (perception of pain evoked by innocuous stimuli) and hyperalgesia (sensitivity to noxious stimuli) that have spread beyond the original site of pain [41, 42]. In migraine, a primary headache, spread of cutaneous allodynia from referred pain area on the ipsilateral head to the other side of head and/or the forearms has already been demonstrated [36, 42]. Sensitization of third-order trigeminovascular neurons that receive convergent input from second-order dorsal horn neurons located in the trigeminal nucleus caudalis (i.e., process sensory information from the head) and the cervical enlargement (i.e., process sensory information from the upper limbs) has been suggested as a pathophysiology of extra-trigeminal spread of cutaneous allodynia during migraine attack [36]. In line with this, long-lasting hyperexcitability of sensory neurons of the rat posterior thalamic nucleus to innocuous and noxious stimulation of the paw was identified by chemical stimulation and sensitization of the cranial dura [42]. In migraine patients, functional MRI assessment of blood oxygenation level-dependent (BOLD) signals showed that brush and heat stimulation at the skin of the hand dorsum produced larger BOLD responses in the posterior thalamus of subjects undergoing a migraine attack with extracephalic allodynia than the corresponding response registered when the same patients were free of migraine and allodynia [42].

Another pathophysiological explanation for the extracephalic, generalized extension of referred pain beyond the trigeminal area involves more rostral Sp5C neurons and brainstem pain modulating structures as well as sensitization of TCC neurons located in the caudal Sp5C.

In animal models of chronic orofacial neuropathic pain involving lesions within peripheral or central components of the trigeminal somatosensory system, alterations in central nervous system (CNS) components of the trigeminal nociceptive system such as the trigeminal brainstem sensory nuclear complex (TBSNC) and associated behavioral changes that occur following trigeminal nerve injury have been reported [31, 43]. Injury to trigeminal nerve branches, such as the inferior alveolar nerve, administration of orofacial inflammatory drugs, or injection of analgesic chemicals into orofacial tissues can cause alterations in the trigeminal primary afferent nerve, which can lead to peripheral sensitization, a hyperexcitable state of the afferent nerve [31, 43, 44]. Changes in the excitability of trigeminal primary afferents following nerve injury (or inflammation) might not be limited to damaged afferents. Local sprouting of adjacent, intact afferents and neuron-glial interaction involving the release of chemical mediators and changes in signaling mechanisms can result in spread of hypersensitivity in trigeminal ganglions and extraterritorial secondary hyperalgesia [31, 43, 44].

Nociceptive transmission from central endings of trigeminal primary afferents to neurons in the TBSNC may take place in rostral components of the TBSNC (e.g., subnucleus oralis, Sp5O). However, it is especially evident in its more caudal components, namely, medullary dorsal horn (MDH, also known as subnucleus caudalis, Sp5C) and the caudalis-interpolaris (Sp5C-Sp5I) transition zone, as well as in upper cervical dorsal horns [31, 42–45]. Several mediators, receptors, and signaling processes have been found to be crucially involved in central sensitization in the TBSNC. These include glutamatergic, neurokinin (e.g., substance P; CGRP) and purinergic (e.g., ATP) mediators released from primary afferents, intracellular signaling processes such as nitric oxide and p-ERK, as well as cytokines from glial cells [31, 43–45]. Central sensitization of the TSNBC has been demonstrated by increased expression of p-ERK and c-Fos and electrophysiologic hyperactivity of the MDH and adjacent regions [31, 43–45].

Following development of trigeminal central sensitization of rat MDH with intramuscular injection of inflammatory irritant, the extensive expansion of mechanoreceptive field has been demonstrated in addition to increased response to noxious stimuli and spontaneous activity [43, 44]. This allodynic response to pinch, tactile, and deep pressure components of receptive fields can extend to the ipsilateral extraterritorial trigeminal area as well as contralateral facial and cervical regions [45, 46]. This kind of extraterritorial sensory abnormality and spread of sensitivity (ETSS) has been confirmed not only in widespread areas innervated by trigeminal nerves but also in extracephalic areas innervated by spinal nerves [43, 47, 48]. Central mechanisms are considered to underlie ETSS extending to contralateral tissues or more wide spread areas [43]. Unilateral injury of trigeminal nerve branches in the inferior alveolar nerve can result in neuropathic pain-like behaviors including extension of ETSS extends beyond ipsilateral orofacial tissues innervated by the nerve that have been injured [46]. These behavioral changes are accompanied by immunohistochemical changes in neurons and glia that are widespread in the MDH, extending behind the MDH region normally representing territory of an injured nerve [43]. Electrophysiological alternations in both wide dynamic range and nociceptive-specific neurons reflecting central sensitization include neuronal receptive fields that have expanded beyond the innervation territory of the injured nerve. They may encompass afferent inputs from extraterritorial trigeminal divisions [43, 45, 46].

The reverse of the previously described situation might also occur, where facial hypersensitivity might occur after spinal nerve injury in an animal pain model [43]. Unilateral injury to upper cervical spinal nerves or inflammation of tissues innervated by spinal nerves can produce bilateral facial hypersensitivity and trigeminal central sensitization associated with extraterritorially expanded neuronal receptive fields [49, 50]. These are consistent with clinical reports noted earlier that nociceptive inputs to the CNS from tissues supplied by spinal nerves can induce ETSS to the trigeminal system [43]. ETSS between trigeminal and spinal nociceptive systems suggests that central sensitization and accompanying behavioral changes might occur in different body regions under chronic orofacial pain conditions such as temporomandibular disorders (TMDs) [43]. In addition, neuroplasticity and glioplasticity expressed as central sensitization might occur concomitantly in parts of the CNS involved in processing or modulating nociceptive information related to each of these different regions [43].

Hypersensitivity of nociceptive processing pathways due to central sensitization in TBSNC can simultaneously lead to dysfunction of endogenous pain inhibitory pathways [33, 43]. There are important brain stem pathways of descending modulation (e.g., rostral ventromedial medulla (RVM) or subnucleus reticularis dorsalis (SRD) in reticular formation) that receive trigeminal nociceptive inputs and send modulatory influences to area of the spinal cord (e.g., spinal dorsal horn) involved in nociceptive transmission from the body [43, 51]. This could explain how centrally sensitized neurons of the TBSNC can cause dysfunction of brain stem pain modulating system, resulting in disturbance of spinal nociceptive circuits and related behavior [43, 51, 52].

In an electrophysiological study of the rat RVM, noxious heating of the tail or perioral skin increased the activity of ON-cells and decreased the activity of OFF-cells before tail flick and jaw-movement responses [51]. Likewise, mustard oil injection into the temporomandibular joint had similar effects on RVM neurons [51]. These findings suggest that RVM ON- and OFF-cells play an important role in the elaboration of diverse nociceptive behaviors evoked by noxious stimulation of widely separated regions of the body [51]. Dysfunction of the endogenous pain inhibitory system in the brain stem has been reported in diseases such as fibromyalgia, TMD, and atypical facial pain, in which central sensitization is regarded as the main pathophysiology [43, 53–55]. Thus, the presence of central sensitization in one region of the body might be a risk factor predisposing to a more generalized central sensitization associated with pain developing in other regions of the body [43].

### 3.4. Diagnostic Difficulty in Greater Occipital Nerve Entrapment

The diagnosis of GON entrapment is clinical. At first glance, diseases involving the GON may be thought to manifest clinically as occipital neuralgia. However, paroxysmal stabbing pain of ON is not the only symptom of GON entrapment. GON entrapment commonly induces continuous aching, tightening, and pressure-like pain in the GON distribution, as well as intermittent stabbing pain in typical ON [7–9]. In addition, chronic GON entrapment not only causes pain in the temporo-occipital and posterior neck, which are the GON distribution areas, but also causes referred trigeminal pain in the facial trigeminal area. GON compression/entrapment has been thought to contribute to the extracranial pathophysiology of occipital headaches and migraines [56]. Therefore, if GON compression/entrapment can be confirmed through imaging tests, especially MR neurography, it will be of great help in diagnosis of GON entrapment.

One study using MR neuroimaging of the GON suggested that MR neurography could be used reliably to diagnose GON neuropathy in patients with unilateral occipital migraine and that there was a good correlation between imaging findings and clinical symptoms [57]. However, their study was limited to only a small cohort of 18 people. It is also unclear whether the disease studied was occipital neuralgia or occipital migraine. According to current classification of headache, headaches are not classified by location, so the term “occipital migraine” is not accepted. Additionally, it is not explained whether the symptoms of the study patients improved through GON decompression and were due to unilateral occipital migraine [57]. In fact, the authors performed 3T MR neurography on more than 500 cases of GON entrapment patients, but we are not confident about the sensitivity and specificity of MR neurography in the diagnosis of GON entrapment. Despite these shortcomings, future advances in MR neurological technology are expected to be helpful in diagnosing GON entrapment.

## 4. Conclusions

At first glance, various pains in the present case, namely, pain in the occipital and neck, especially in the jaw, eyes and cheeks, pain in the ears, and pain in the limbs, did not seem to be related to each other. However, these symptoms could be understood when considering occurrence of TCC sensitization and hypersensitivity by GON entrapment based on convergence of the trigeminal nerve and occipital nociceptive afferents into MDC and TCC. In addition, considering that pain occurring in the ear ultimately integrates into the caudal trigeminal nucleus, pain in the ear is believed to have the same mechanism as trigeminal referred pain. Pain radiating from the back of the head to the neck and shoulders can also be understood by considering pain distribution area of the GON. Therefore, pain projection and association areas in GON entrapment are much more diverse than we thought, covering a wide range including the head, neck, and face.

The most difficult pain to describe is referred pain in the lower back and extremities. This pain also improved after GON decompression. Two pathophysiologies may be involved in this phenomenon. First, generalized pain might be caused by sensitization of third-order nociceptive neurons in the thalamus. Second, spontaneous pain might occur throughout the body due to dysfunction of the descending brain stem pain-modulating pathway caused by sensitization and hyperexcitation of the MDH and TBSNC.

## Data Availability

No data were used to support this study.
